# Supplementary material for: The emergence of a novel CA1 spatial map requires direct entorhinal input
Source: bioRxiv. 2026 Jul 7:2026.07.02.736055. Preprint. [Version 1] doi: 10.64898/2026.07.02.736055 (PMC13370420; doi:10.64898/2026.07.02.736055)
Supplement: Supplement 1 [file NIHPP2026.07.02.736055v1-supplement-1.pdf]

bioRxiv preprint doi: https://doi.org/10.64898/2026.07.02.736055; this version posted July 7, 2026. The copyright holder for this preprint (which was not certified by peer review) is the author/funder, who has granted bioRxiv a license to display the preprint in perpetuity. It is made available under a CC-BY 4.0 International license.

633 # Supplementary Figures

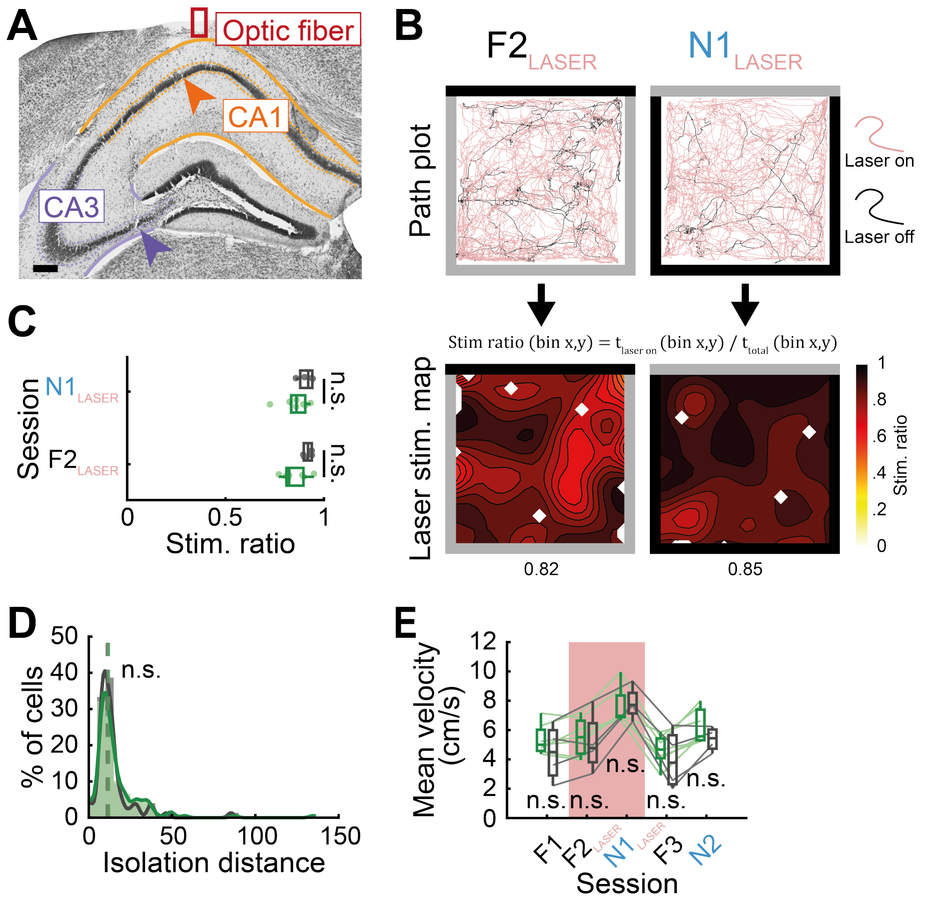

Figure S1. Hippocampal recordings and optogenetic manipulation in freely exploring mice

(A) Nissl-stained coronal section showing an example optic fiber tract (red rectangle) and tetrode recording sites in CA1 (orange arrow) and in CA3 (purple arrow). Scale bar: 200µm. **(B)** Optogenetic stimulation strategy. Top: example path plots during familiar and novel environments sessions with laser stimulation. Red and black paths represent periods with the laser on and off, respectively. To quantify stimulation coverage, we computed a stimulation ratio for each spatial bin (2cmx2cm) as the fraction of the time with laser on relative to the total time. Bottom: contour plots of stimulation ratios across familiar and novel environments. Values below each plot represents arena-wide mean stimulation ratios. **(C)** Average stimulation ratios for both Opsin (n=7) and Control (n=4) animals. F2: p=0.0936; N1: p=0.1843, permutation test. **(D)** Distribution of unit isolation distances for all recorded units from the Opsin (N=168) and Control (N=75) groups. p=0.3286, Wilcoxon rank-sum test. **(E)** Mean running velocity across all sessions for all Opsin (n=7) and Control (n=4) animals. F1: p=0.3413; F2: p=0.6767; N1: p=0.7221; F3: p=0.4713; N2: p=0.2296, permutation test.

Box plots show median and interquartile range. Dashed vertical lines overlaying histograms show medians. Green, Opsin; gray, Control.

bioRxiv preprint doi: https://doi.org/10.64898/2026.07.02.736055; this version posted July 7, 2026. The copyright holder for this preprint (which was not certified by peer review) is the author/funder, who has granted bioRxiv a license to display the preprint in perpetuity. It is made available under a CC-BY 4.0 International license.

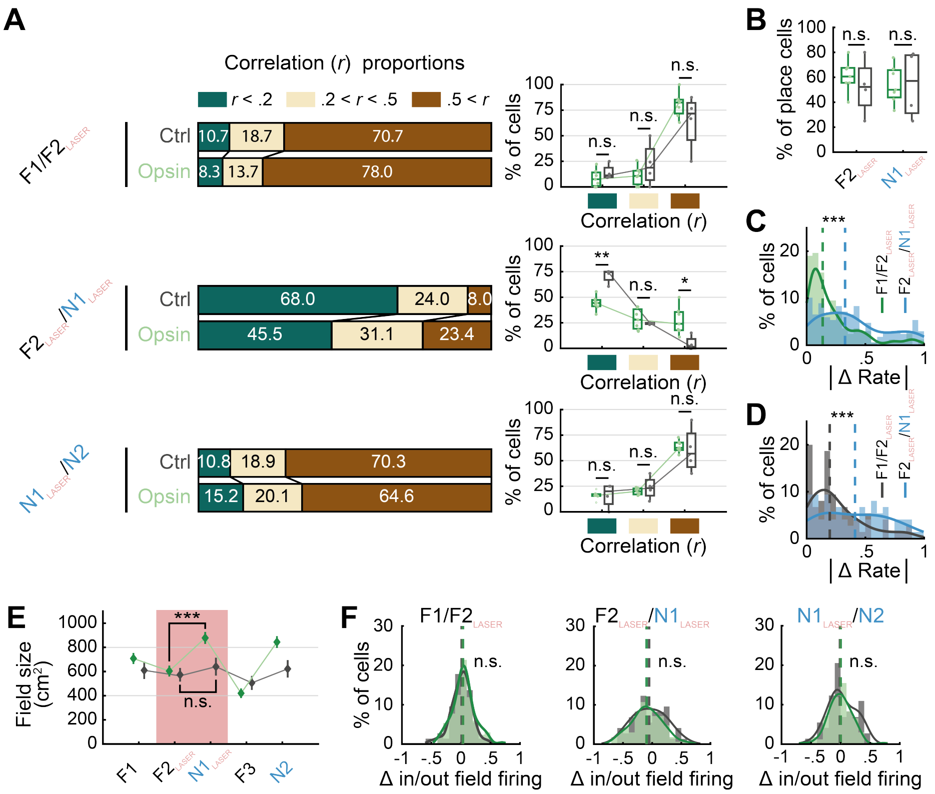

Figure S2. The effect of ECIII input inhibition on place cell firing properties in a familiar and novel environment.
(A) Left: proportions of Opsin (*N*=168) and Control (*N*=75) place cells with low (green, $r < 0.2$), intermediate (beige, $0.2 < r < 0.5$) and high (brown, $r > 0.5$) Pearson correlation coefficients between sessions F1 and F2 (top), F2 and N1 (middle) and, N1 and N2 (bottom). Right: average proportions per animal (Opsin, *n*=7; Control, *n*=4). Between sessions F1 and F2 (top). $r < 0.2$, p=0.2960; $0.2 < r < 0.5$, p=0.2658; $r > 0.5$, p=0.1963. Between sessions F2 and N1 (middle). $r < 0.2$, **p=0.0030; $0.2 < r < 0.5$, p=0.4260; $r > 0.5$, *p=0.0181. Between sessions N1 and N2 (top). $r < 0.2$, p=0.9698; $0.2 < r < 0.5$, p=0.4804; $r > 0.5$, p=0.6798. Permutation test. **(B)** Fraction of place cells for Opsin (*n*=7) and Control (*n*=4) animals during familiar (F2) and novel (N1) sessions with ECIII input inhibition. For session F2, p=0.4290; for session N1, p=0.9799. Permutation test. **(C)** Distribution of place cell absolute firing rate change for the Opsin group (*N*=168) between familiar sessions without (F1) and with (F2) ECIII input inhibition (green) and between familiar (F2) and novel (N1) sessions with ECIII input inhibition (blue). ***$p=5.1 \times 10^{-13}$, Wilcoxon signed-rank test. **(D)** Same as **(C)** for the Control group (*N*=75). ***$p=1.7 \times 10^{-4}$, Wilcoxon signed-rank test. **(E)** Average place field size (median±SEM) for Opsin and Control groups across sessions. Between sessions F2 and N1: Opsin group (F2, *N*=140 place fields; N1, *N*=132 place fields): ***$p=3.3 \times 10^{-4}$; Control group (F2, *N*=60 place fields; N1, *N*=64 place fields): p=0.0593, Wilcoxon signed-rank test. **(F)** Distribution of in-field versus out-of-field firing rate ratio change. Left: place fields between sessions F1 and F2 (Opsin, *N*=123; Control, *N*=54), p=0.3102, Wilcoxon rank-sum test. Place fields between sessions F2 and N1 (Opsin, *N*=106; Control, *N*=53), p=0.3449, Wilcoxon rank-sum test. Place fields between sessions N1 and N2 (Opsin, *N*=114; Control, *N*=58), p=0.5098, Wilcoxon rank-sum test.
Box plots show median and interquartile range. Dashed vertical lines overlaying histograms show medians. Green, Opsin group; gray, Control group.

bioRxiv preprint doi: https://doi.org/10.64898/2026.07.02.736055; this version posted July 7, 2026. The copyright holder for this preprint (which was not certified by peer review) is the author/funder, who has granted bioRxiv a license to display the preprint in perpetuity. It is made available under a CC-BY 4.0 International license.

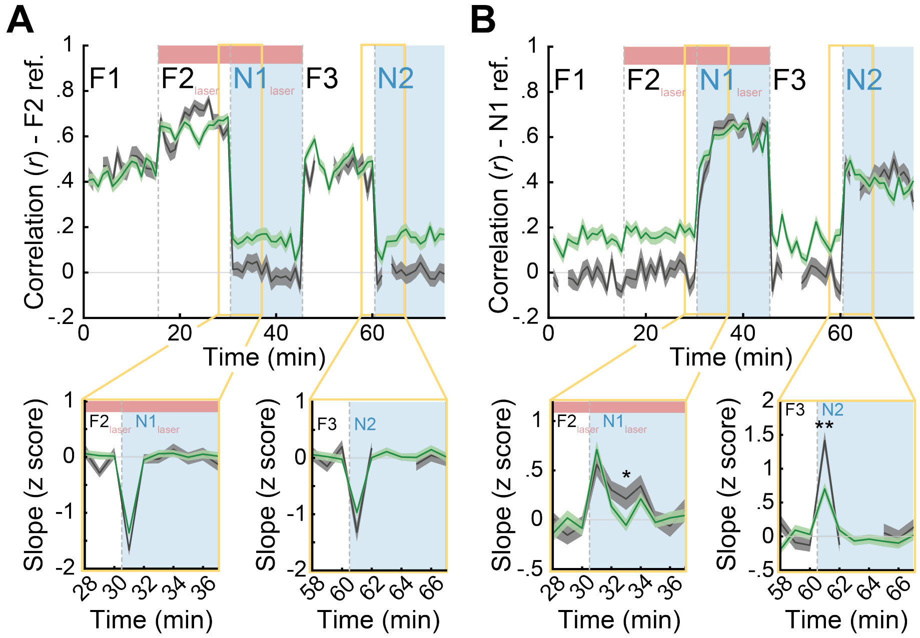

Figure S3. Remapping kinetics

(A-B) Top: Pearson's correlation between 1-minute block rate maps and **(A)** the entire familiar session rate map (F2; reference, ref), and **(B)** the entire first novel session rate map (N1; reference, ref) (Opsin, *N*=168; Control, *N*=75). Bottom: expanded view of the z-scored derivative of the Pearson's correlation shown above, indicating the rate of representational change between consecutive minutes for transitions between familiar and novel environment sessions. All panels show medians±SEM. See Table S1 for statistics.

Green, Opsin group; gray, Control group.

bioRxiv preprint doi: https://doi.org/10.64898/2026.07.02.736055; this version posted July 7, 2026. The copyright holder for this preprint (which was not certified by peer review) is the author/funder, who has granted bioRxiv a license to display the preprint in perpetuity. It is made available under a CC-BY 4.0 International license.

634 # Supplementary Tables

Minute	Session F2 ref. *p*-value	Session N1 ref. *p*-value
1	0.7346	0.0035**
2	0.8395	0.0067**
3	/	/
4	0.6029	0.0034**
5	0.4761	0.0015**
6	0.9740	0.0569
7	0.0615	0.0115*
8	0.8768	0.1497
9	0.4816	0.0019**
10	0.3430	0.0104*
11	0.3595	0.0018**
12	0.4487	0.0043**
13	0.3859	0.1276
14	0.7362	0.0001***
15	0.8805	0.0074**
16	0.4755	4.5x10-5***
17	0.2683	0.0016**
18	0.0838	0.0008***
19	0.6821	0.1919
20	0.9766	0.0110*
21	0.4954	0.0220*
22	0.1771	0.0038**
23	0.0014**	0.0615
24	0.0002***	5.6x10-5***
25	0.0396*	0.0037**
26	0.0101*	0.0062**
27	0.4006	0.0023**
28	0.6622	0.0063**
29	0.0484*	0.0001**
30	0.2815	0.0029**
31	0.0065**	0.0029**
32	0.0246*	0.3608
33	0.0047**	0.9934
34	0.0099**	0.6240
35	0.0165*	0.1146
36	0.0225*	0.2092
37	0.0006***	0.1508
38	0.0103*	0.7277
39	0.0001***	0.7824
40	0.0058**	0.7353
41	0.0023**	0.1513
42	0.0350*	0.2957
43	0.0145*	0.8758
44	0.5918	0.0803
45	0.0125*	0.4411
46	0.7163	0.0001**
47	0.0144	0.1599
48	0.1046	6.3x10-5***
49	/	/

bioRxiv preprint doi: https://doi.org/10.64898/2026.07.02.736055; this version posted July 7, 2026. The copyright holder for this preprint (which was not certified by peer review) is the author/funder, who has granted bioRxiv a license to display the preprint in perpetuity. It is made available under a CC-BY 4.0 International license.

50	/	/
51	0.2481	0.0090**
52	0.7457	0.0595
53	0.4218	0.0812
54	0.7950	0.0476*
55	0.4594	0.0165*
56	0.2120	0.0070**
57	0.7398	0.0042**
58	0.2842	0.3714
59	0.0549	0.0013**
60	0.6669	9.3x10-5***
61	0.0023**	0.4502
62	0.2820	0.2733
63	/	/
64	0.0297*	0.3652
65	0.0025**	0.7725
66	0.0020**	0.9680
67	0.0002***	0.5047
68	0.0007***	0.5141
69	0.0064**	0.3033
70	0.1233	0.1045
71	0.0170*	0.0009***
72	1.7x10-5***	0.8949
73	0.0015**	0.0556
74	0.0019**	0.2399
75	0.0009***	0.0959

Table S1. Statistical comparisons of Pearson's correlation between Opsin and Control groups for each 1-minute block with F2 and N1 as reference sessions. *$p < 0.05$; **$p < 0.01$; ***$p < 0.001$

**LIF Parameters**		**Simulation Parameters**	
**Parameter**	**Value**	**Parameter**	**Value**
E_L^k	-65	τ_{inh}	0.1
R^k	10	Δt_{plast}	1
V_{th}^k	-45	β	0.01
$w^{\text{IP}_{\text{SST}}}$	$5000/N_{\text{CA1}}$	σ_{pf}	6
$w^{\text{PI}_{\text{SST}}}$	$75/N_{\text{SST}}$	Δt	0.001
$w_0^{\text{IP}_{\text{PV}}}$	$750/N_{\text{CA1}}$	v_0	20
$w_0^{\text{PI}_{\text{PV}}}$	$100/N_{\text{PV}}$	a	0.3
τ^{A}	1	m^{EC}	800
τ^{B}	0.25	m^{CA3}	30
τ^{PV}	0.1	η	15
τ^{SST}	0.1	α	0.25
		η_{inh}	150
		L	50
		t_N	250
		N_{CA3}	169
		N_{CA1}	289
		N_{SST}	30
		N_{PV}	30

Table S2. Model parameters. These parameter settings were applied consistently across all simulations, unless explicitly stated otherwise.
